# Design and Verification of a Dry Sensor-Based Multi-Channel Digital Active Circuit for Human Brain Electroencephalography Signal Acquisition Systems

**DOI:** 10.3390/mi10110720

**Published:** 2019-10-25

**Authors:** Chin-Teng Lin, Chi-Hsien Liu, Po-Sheng Wang, Jung-Tai King, Lun-De Liao

**Affiliations:** 1 Centre for AI, Faculty of Engineering and Information Technology, University of Technology Sydney, Sydney NSW 2007, Australia; 2 Brain Research Center, National Chiao Tung University, Hsinchu 300, Taiwanl.swang@hotmail.com (P.-S.W.); jtchin2@gmail.com (J.-T.K.); 3 Institute of Electrical and Control Engineering, National Chiao Tung University, Hsinchu 300, Taiwan; 4 Institute of Biomedical Engineering and Nanomedicine, National Health Research Institutes, Miaoli 35053, Taiwan

**Keywords:** electroencephalography (EEG), brain–computer interface (BCI), dry sensor, event-related potential (ERP)

## Abstract

A brain–computer interface (BCI) is a type of interface/communication system that can help users interact with their environments. Electroencephalography (EEG) has become the most common application of BCIs and provides a way for disabled individuals to communicate. While wet sensors are the most commonly used sensors for traditional EEG measurements, they require considerable preparation time, including the time needed to prepare the skin and to use the conductive gel. Additionally, the conductive gel dries over time, leading to degraded performance. Furthermore, requiring patients to wear wet sensors to record EEG signals is considered highly inconvenient. Here, we report a wireless 8-channel digital active-circuit EEG signal acquisition system that uses dry sensors. Active-circuit systems for EEG measurement allow people to engage in daily life while using these systems, and the advantages of these systems can be further improved by utilizing dry sensors. Moreover, the use of dry sensors can help both disabled and healthy people enjoy the convenience of BCIs in daily life. To verify the reliability of the proposed system, we designed three experiments in which we evaluated eye blinking and teeth gritting, measured alpha waves, and recorded event-related potentials (ERPs) to compare our developed system with a standard Neuroscan EEG system.

## 1. Introduction

A brain-computer interface (BCI) is an interface/communication system that translates brain signals into machine commands [1]. One of the goals of the BCI technology is to provide communication and control capacities to people with severe motor disabilities [2]. BCIs are communication systems that can recognize a user’s commands from their brainwaves alone and then react to these commands [3]. In recent BCI research, several approaches, such as electroencephalography (EEG), magnetoencephalography (MEG), and functional magnetic resonance imaging (fMRI), have been used to acquire brain activity signals [4]. Unfortunately, the large size, high price, and complexity of the required recording equipment, such as the Neuroscan EEG system, limit the practical application of these methods in daily life.

The use of EEG signals has become the most common approach in BCI research because these signals offer high usability and reliability. BCIs can be divided into two categories according to how brain activity is acquired [5]: invasive BCIs and noninvasive BCIs [6,7]. Because of the risks associated with invasive BCIs, we mainly focus on noninvasive EEG-based BCI methods here. The first devices to be developed were noninvasive EEG-based BCI devices with traditional wet sensors. However, these traditional sensors are uncomfortable to wear and have other disadvantages, such as requiring the user to apply a conductive gel and preparing the skin. Therefore, a significant goal for future BCI devices is to develop a means of acquiring EEG signals in a comfortable and convenient manner [5].

The development of EEG-based BCIs and their corresponding applications has already been introduced above and reviewed in the literature [8]. P300-based BCI systems have also been developed for disabled users [9,10]. The current applications of P300-based BCI systems range from controlling a virtual hand [11] or using a neuroprosthesis [10] to surfing the web [12]. In the past, BCIs have been used to help patients with many different disorders. In recent years, BCI research has focused on the application of BCIs in the daily lives of healthy people [13]. A prosthetic system that can continuously monitor fluctuations in cognitive state in the workplace and/or the home could be beneficial to many patients [14]. However, to be practical for conventional clinical use, such a system must be noninvasive, lightweight, battery-powered, and easy to apply and remove [15].

In addition to the disadvantages of current brainwave monitoring systems in terms of inconvenience, another key drawback is their use of wet electrodes [16]. The conductive gel that must be used with such electrodes eventually dries, reducing the EEG monitoring performance of the system. The use of dry sensors would offer a gel-free solution to this problem [17,18,19], at the cost of increasing both the contact impedance and the effect of cable movement artifacts [16]. While passive dry-sensor EEG acquisition systems have been extensively studied in recent years, a common issue encountered in these systems is that the input impedance is greatly influenced by the long cable due to the long distance that the EEG signals must travel from the scalp to the back-end circuit, making these systems very sensitive to cable artifacts [16].

In this study, we propose an active-circuit EEG acquisition system. Specifically, we designed a lightweight, small-sized, low-cost, and wireless EEG signal acquisition system. An active circuit amplifies and filters raw EEG signals and then digitizes them. An active circuit and a dry sensor together compose an active sensor. To reduce the main sources of interference, including cable motion artifacts and 60 Hz interference, the path from the scalp to the active circuit, a distance that the EEG signals must travel, is minimized. We believe that this active-circuit EEG acquisition system could offer ease of use for both healthy and disabled individuals in daily life in the near future.

## 2. Materials and Methods

### 2.1. Disposable Dry EEG sensors

**Figure 1:**
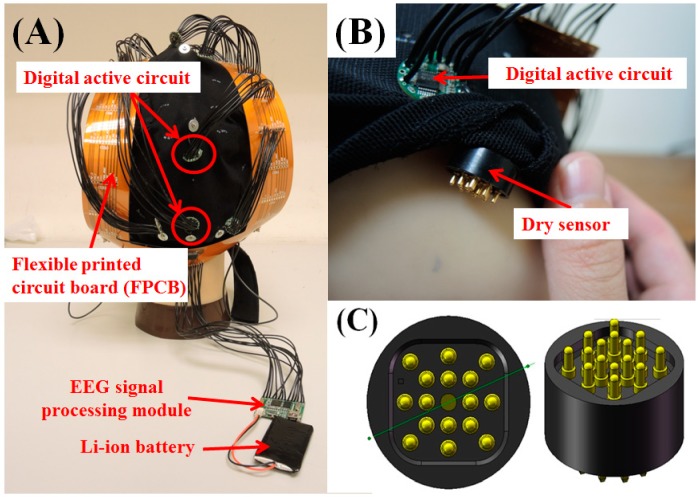
(**A**) The proposed active-circuit system for electroencephalography (EEG) measurement. (**B**) Active circuit and dry sensor. (**C**) Dry sensor design, as we previously reported.

The photographs of our proposed wireless and wearable BCI device, which includes active circuits, dry sensors, a flexible printed circuit board (FPCB), and EEG signal processing units, are shown in Figure 1A,B. The dry EEG sensors are designed to contact the surface of the subject’s scalp via sixteen probes, as shown in Figure 1C. Each probe is constructed from four components: (1) a probe head, (2) a plunger, (3) a spring, and (4) a barrel (Figure 1C). The manufacturing process used to fabricate the dry EEG sensors was reported in detail in our previous publication [17]. We summarize some of the important details of this manufacturing process below.

The spheroid fabricated for each probe head is approximately 1.1 mm in radius. For good conductivity, the heads of the probes are coated with gold (Au) material before being embedded into the plungers, which are made of beryllium copper (BeCu) material. These plungers are optimized by means of turning and plating, resulting in remarkably straight and exact plunger surfaces, which provide the basis for a long operating lifetime. A force buffer of 20 g is applied to each spring before connection to the plunger. The spring is covered by an outside barrel. The spring force of approximately 20 g was selected mainly for consistency with the intended application in human EEG measurements. The spring force must be sufficiently large to ensure that the sensors can adhere to the subject’s scalp surface while enabling a satisfactory sensor–skin contact impedance [15]. Increases in the spring force are designed to be proportional to the spring travel distance (i.e., 2 mm). During the process of probe assembly, the spring is compressed by a certain working distance. This preloading ensures that some amount of force is applied from the beginning of the contact process and that the plunger is completely pushed back once it contacts the scalp surface.

The sixteen probes are then inserted into a thin Cu plate, which serves as the flexible substrate for the sensor. This substrate is constructed as a thin circular plate (Cu) with a thickness of 1.5 mm and a diameter of 12 mm. The probes embedded in the thin plate are collectively conductive after insertion. In addition, the thinness of the plate endows it with flexible substrate characteristics when force is applied. This flexible substrate achieves good conformability with the scalp, especially when force is applied to the sensor. Buffering effects are provided by both the probes and the flexible substrate, enabling the sensor to achieve good contact with the measurement surface with minimal discomfort when force is applied. Finally, the probes and the flexible substrate are placed in a mold for an injection-molding process, during which the sensor is encased in a soft silicone material that is injected into the mold [15].

### 2.2. Active Circuit

EEG signals measured from the scalp range from 10 to 100 µV; hence, because these signals are relatively weak, they are rather difficult to measure in the presence of noise. The proposed active-circuit EEG signal acquisition system with dry sensors was designed to acquire these weak signals without noise. The active circuit is an EEG signal processing circuit. First, an amplifying circuit with a high total gain is used to amplify small EEG signals on the order of 10–150 µV [20]. Second, to reduce the loading effect on the sensors, the circuit should have a high input impedance. Third, because the frequency range of EEG signals is approximately 0.5–100 Hz, a bandpass filter with a narrow bandwidth is needed to filter out the DC offset and high-frequency noise. Finally, we digitize the EEG signals for digital transmission.

**Figure 2:**
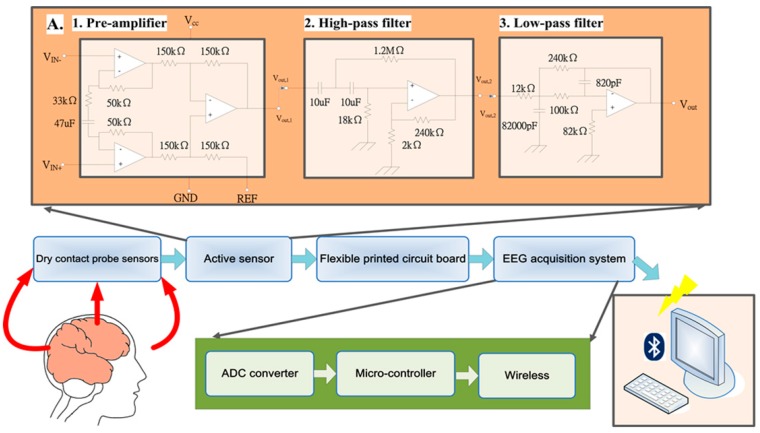
Overview of the hardware framework of the developed system. The electroencephalography (EEG) signals are measured by dry sensors, and the signals are then amplified and filtered by active circuit units. The amplified EEG signals are transmitted via cables to the EEG signal acquisition system, which digitizes the signals and transmits them to a personal computer.

Because of its high input impedance, the preamplifier must be an instrumentation amplifier [21]. The second-stage amplifier must be a high-pass filter because the DC offset needs to be filtered out before the EEG signal is amplified. The third-stage amplifier should be a low-pass filter to filter out high-frequency noise, thus completing the bandpass filter. Finally, we include an analog-to-digital converter (ADC). The signal flow chart is shown in Figure 2.

In the first stage, an instrumentation amplifier (INA333, Texas Instruments, Dallas, TX, USA) is used due to its extremely high input impedance (100 GΩ) and high common-mode rejection ratio (CMRR) (100 dB). In addition, the INA333 amplifier requires low power consumption and is small in size. These advantages make the INA333 amplifier ideal for our portable system. The transfer function of the designed preamplifier circuit is shown in Equation (1):

(1) $$G=1+\left(\frac{100\;k\Omega }{33\;k\Omega +\frac{1}{jw47\mu F}}\right)$$

The gain of the preamplifier unit is set to 4.03 V/V, and the cut-off frequency for the signal passed to the high-pass filter is 0.103 Hz.

In the second and third stages, a CMOS operational amplifier (OPA2333, Texas Instruments, Dallas, TX, USA) is used to complete the bandpass filter. The OPA2333 amplifier has a low-offset voltage (10 µV max), near-zero drift (0.05 μV/°C max) with time and temperature, a low quiescent current (17 μA) and a micropower operational amplifier.

The OPA2333 amplifier offers an excellent CMRR (130 dB) without the crossover associated with traditional complementary input stages. This design results in superior performance in driving the ADC without degradation of differential linearity. The EEG signals amplified by the preamplifier are first filtered by the high-pass filter with a 0.108 Hz cut-off frequency in the second stage. The gain of the high-pass filter is set to 121 V/V. The third stage includes a low-pass filter, which is also an inverting amplifier. The gain of the low-pass filter is set to −20 V/V, and the cut-off frequency is 125.3 Hz. In this digitization stage, an ADS1291 ADC (Texas Instruments, Dallas, TX, USA) is used. The ADS1291 is a low-power, high-resolution (24 bits) single-channel delta–sigma ADC designed for biopotential measurements. Overall, the total gain of the active circuit is approximately 9752 V/V, and the circuit filters out noise below 0.103 Hz and above 125 Hz.

Finally, the amplified and filtered EEG signals are digitized by the ADC (ADS1291, Texas Instruments, Dallas, TX, USA). A total of 8 channels of active circuits are operated at approximately 2.8 mA with a 3 V DC power supply. Each active circuit has a small circular shape with a diameter of 16 mm.

### 2.3. Electroencephalography (EEG) Signal Processing Module

As shown in Figure 2, the EEG signal processing module consists of four major units: (1) a front-end ADC converter unit, (2) a microcontroller unit, (3) a wireless unit, and (4) a power management unit. For the ADC unit, an ADS1298 (Texas Instruments, Dallas, TX, USA) is used. The ADS1298 integrates 8 high-resolution (24 bits) delta–sigma ADC channels and a programmable gain amplifier (PGA) and provides a high data rate (32 ksps (samples per second)). The ADS1298 also has the features of low power consumption, small size, and low cost. The gain of the PGA of the ADS1298 is set to 1, and the sampling rate is set to 512 Hz and is synchronized with the microcontroller unit via a serial peripheral interface (SPI).

In the microcontroller unit (MSP430F5522, Texas Instruments, Dallas, TX, USA), the sampling rate (512 Hz) is set to be equal to that of the ADC, and the digitized signals are received. Then, the MSP430 transmits the EEG signals to the wireless/Bluetooth unit via a UART interface. The MSP430 is optimized with extensive low-power modes to achieve extended battery life for portable applications. The MSP430 incorporates a 16-bit RISC CPU, peripherals, 10 kB of static random access memory (SRAM), and 128 kB of flash memory. Dedicated embedded emulation logic resides on the device itself and is accessed via joint test action group (JTAG) using no additional system resources.

Bluetooth is a wireless technology standard for transmitting/receiving data over short distances between fixed or mobile devices. Because Bluetooth provides a high level of security and wireless capability and helps overcome issues encountered in the synchronization of these devices, it is integrated into everything from phones to medical devices.

In our system, the HL–MD08R–C2 Bluetooth module is used. The HL–MD08R–C2 is an integrated Bluetooth module that supports a baud rate of 1.2 to 921.6 kbps and uses the CSR BlueCore4–External as the major Bluetooth chip. CSR’s BlueCore4–External is a single-chip radio and baseband IC for Bluetooth 2.402–2.480 GHz systems with enhanced data rates (EDRs) of up to 3 Mbps. When used with the Cambridge silicon radio (CSR) Bluetooth software stack, the BlueCore4–External provides a Bluetooth system that is fully compliant with the v2.0 specifications for data and voice communications. All hardware and device firmware of the HL–MD08R–C2 are fully compliant with the Bluetooth v2.0 + EDR specifications.

The power management circuit in our EEG signal acquisition system includes two components: (1) a power supply circuit and (2) a charging circuit. In our EEG signal acquisition system, the operating voltage Vcc is 3 V, and the virtual ground of the analog circuit is at 1.5 V. To provide stable voltages of 1.5 V and 3 V, an LP3985 regulator is used to regulate the battery voltage to 3 V. The LP3985 is a micropower, low-dropout, and ultralow-noise 150 mA CMOS voltage regulator capable of supporting a maximum output current of 550 mA.

**Figure 3:**
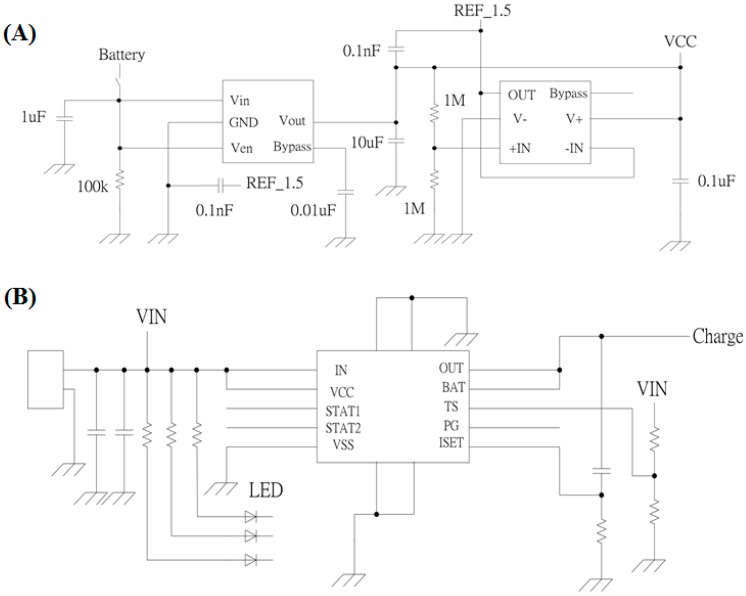
(**A**) Power supply circuit and (**B**) charging circuit in the electroencephalography (EEG) signal acquisition system.

Furthermore, the turn-on time can reach 200 μs. A voltage divider circuit is used to divide the 3 V voltage into 1.5 V, and a unity amplifier constructed from an AD8628 is used to provide a voltage buffer. The entire power supply circuit is shown in Figure 3A.

The BQ24010DRC charging circuit includes an integrated power field effect transistor (FET) and a current sensor for 1 A charging applications. The maximum charging current reaches 1 A. The battery power is automatically detected by the charging circuit, and the circuit will switch to charging mode when the battery power is not sufficiently high. The BQ24010DRC also protects the battery from overcharging and overdriving. The charging circuit is shown in Figure 3B.

**Figure 4:**
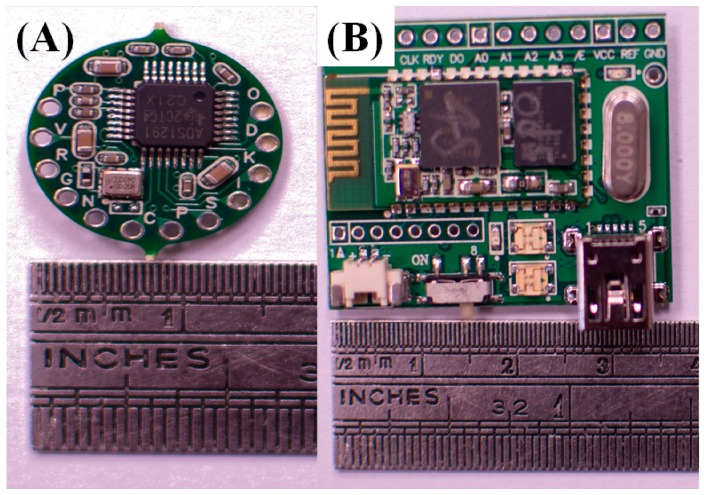
(**A**) An active circuit and (**B**) an electroencephalography (EEG) signal processing module.

This module is operated at 33 mA (including eight active circuits) with a 3.7 V DC power supply and can continuously function for over 20 h with a commercial 650 mAh Li–ion battery. The total size of the EEG signal acquisition system is approximately 36 × 28 mm^2^. Because of its microscale design, the charging circuit can be embedded in our system without causing discomfort. There are nineteen leads in our EEG signal acquisition module for the sixteen channels, the reference, the power Vcc, and the virtual ground of the front-end analog circuit. The electrodes, which are connected to the leads corresponding to the virtual ground, and the EEG reference are placed behind the user’s left and right ears, respectively. The Vcc lead is connected to the active circuit to provide power. An active circuit and an EEG signal processing module are shown in Figure 4A,B, respectively. The specifications of the EEG signal acquisition system are listed in Table 1.

### 2.4. Flexible Printed Circuit Board

**Figure 5:**
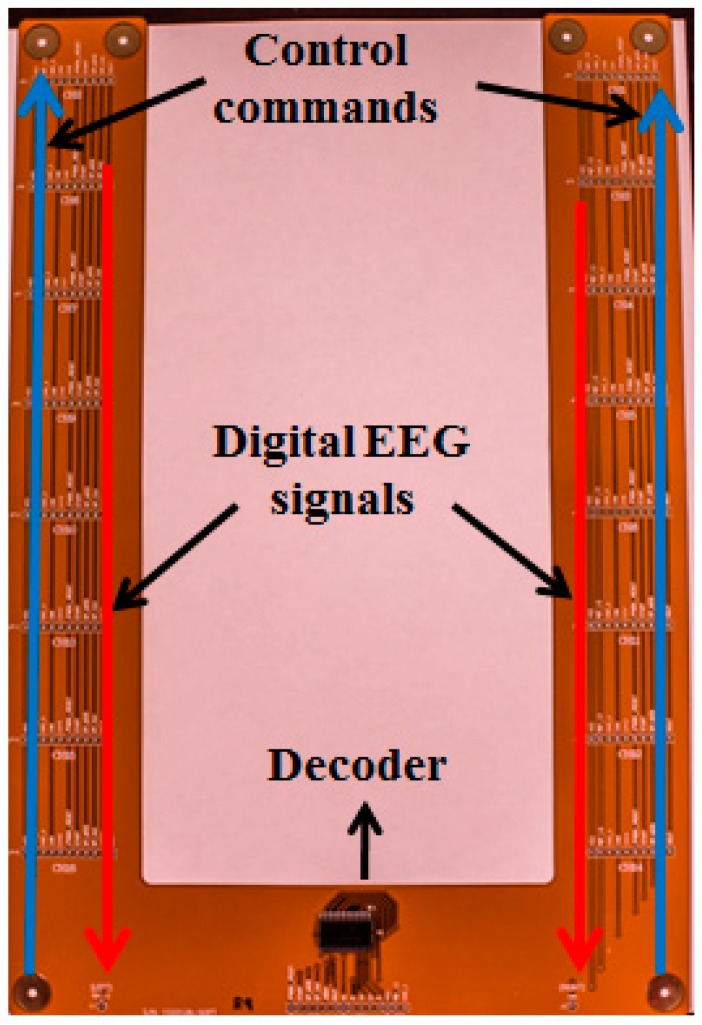
Top view of the flexible printed circuit board (FPCB). The red arrows indicate the direction of the digital electroencephalography (EEG) signals, and the blue arrows indicate the direction of the control commands.

The FPCB is the connection between the active sensors and the EEG signal processing module, as shown in Figure 1A and Figure 5. The FPCB includes a decoder for communication between the active sensors and the EEG signal processing module. The microcontroller in the EEG signal processing module sends orders and receives data from a specific ADC channel among the active circuits through the decoder in the FPCB, which can help the microcontroller select a specific active sensor.

### 2.5. Experiments for Verification

The experiments reported in this paper were approved by the Institutional Review Board (IRB) of the National Chiao Tung University (NCTU–REC–106057) and were carried out in accordance with the rules of the Declaration of Helsinki. Three experiments were designed to test and verify the 8-channel active-circuit EEG signal acquisition system: (1) eye blinking and teeth gritting, (2) eyes-open/eyes-closed states, and (3) event-related potentials (ERPs). In EEG research, experiments evaluating the signals during ERP acquisition are commonly performed with the opening and closing of the eyes [22].

## 3. Results and Discussion

### 3.1. Eye Blinking and Teeth Gritting

**Figure 6:**
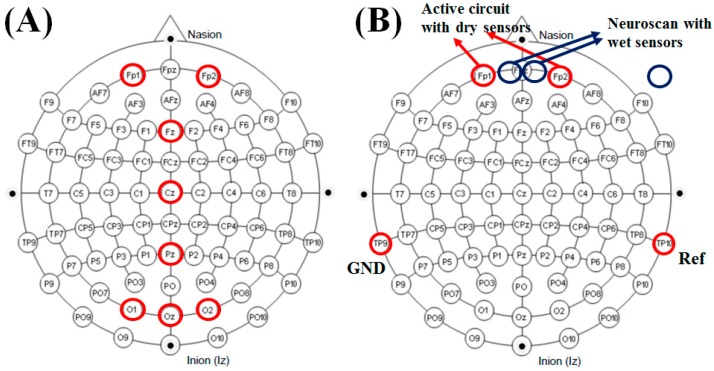
(**A**) Dry sensor placement for the 8-channel active-circuit electroencephalography (EEG) signal acquisition system. (**B**) Electrode placement in the eyes-open/eyes-closed experiment.

**Figure 7:**
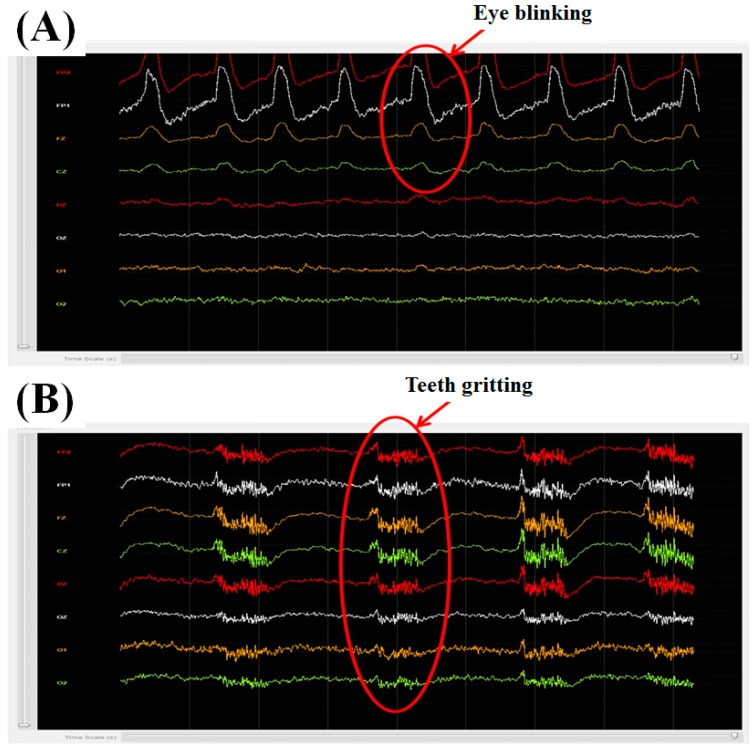
(**A**) Eye blinking and (**B**) teeth gritting tests.

According to the equivalent circuit model for EEG measurement [23], the impedance between the skin and the developed dry sensor ranged from 4 to 14 kΩ, based on our experiments. The impedance of the developed dry sensor herein ranged from 4 to 14 kΩ, which is in the proper range for detecting EEG signals. We performed two general tests: eye blinking and teeth gritting. The computer interface of the 8-channel active-circuit EEG acquisition system was used to display EEG data on the monitor in real time. We mark the electrode placements of the 8-channel active-circuit system on the well-known 10-20 EEG system diagram in Figure 6A. The subject was requested to blink his or her eyes (Figure 7A) and grit his or her teeth (Figure 7B), and the corresponding EEG signals were monitored in real time on the screen.

### 3.2. Eyes-Open/Eyes-Closed States

Alpha waves are a type of brain wave that ranges in frequency from 8 to 13 Hz. The alpha waves obviously increase in amplitude during relaxation with closed eyes. In contrast, the alpha waves are decrease when the eyes are open. This well-known phenomenon can be used as a metric for preliminary evaluation of EEG recordings. Two subjects participated in this experiment. The placement of the electrode used in this experiment is shown in Figure 6B. Both subjects were male and aged 23 or 24 years old. Each subject participated in two periods of testing. During the first period, the subject was asked to open their eyes (normal state) for 30 s. Then, in the second period, the subject was asked to close their eyes for 30 s.

**Figure 8:**
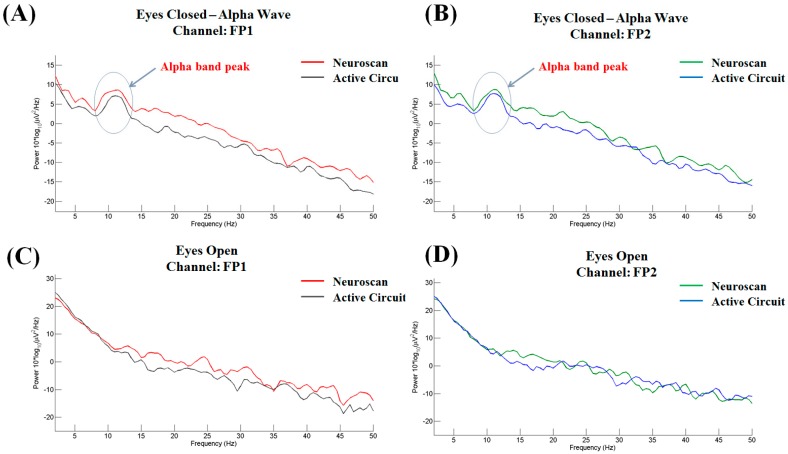
Power spectra in the eyes-closed condition: (**A**) FP1 and (**B**) FP2. Power spectra in the eyes-open condition: (**C**) FP1 and (**D**) FP2.

The eyes-closed results for subject 1 are shown in Figure 8A,B for channels FP1 and FP2, respectively, and the eyes-open results are shown in Figure 8C,D for channels FP1 and FP2, respectively. In contrast to the results obtained in the eyes-open condition, the power spectra observed in the eye-closed condition exhibit a large peak at approximately 8–13 Hz, which corresponds exactly to the alpha band.

### 3.3. Event-Related Potentials

**Figure 9:**
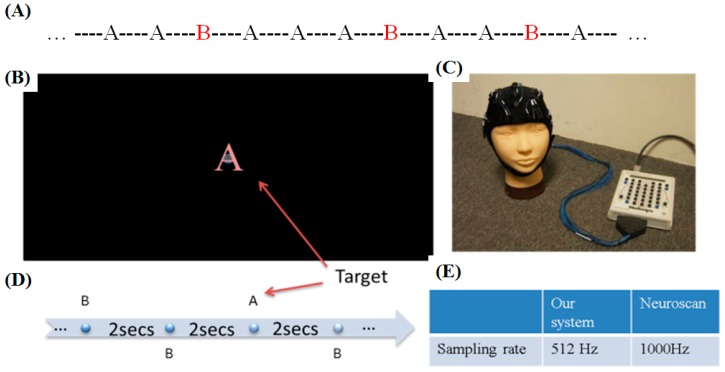
(**A**) A series of random stimuli in the oddball task. (**B–E**) Settings for the event-related potentials (ERP) experiment.

ERPs are a response to brain stimulation and can be measured on the scalp [22]. ERPs reflect brain electrical activity that is related to cognitive stimuli. The P300 potential is a relatively large positive waveform that peaks with a latency of 300–900 ms and is widely used in cognitive science and cognitive psychology research [22]. The oddball protocol, which employs frequent and infrequent stimuli, is a general ERP experiment used to stimulate the P300 component [24]. Two stimuli are presented randomly, as shown in Figure 9A, and the subject is requested to discriminate the infrequent stimulus from the frequent standard stimulus. The P300 component is stimulated as a reaction to the infrequent target stimulus.

In this study, two different stimuli were presented: “A” and “B”. For the oddball task, the screen presented both frequent standard and infrequent stimuli to the subject. For example, “B” might be presented as the frequent stimulus and “A” as the infrequent stimulus, as shown in Figure 9A. Each stimulus was presented in the center of the screen, as shown in Figure 9B.

The oddball experiment included two sections, with each section lasting 10 min. Each stimulus was presented for 0.075 s. During the following 1.925 s, the subject waited for the next stimulus. There were 300 trials in each section, and each trial lasted 2 s (Figure 9D). The probability of the frequent standard stimulus was set to 80%, and the probability of the infrequent target stimulus was set to 20% [17]. In each trial, the stimuli were quickly flashed in the center of the screen, and the screen was blank until the next event.

To verify that the subject’s response was correctly elicited by the target stimulus, the subject was requested to press a button when the oddball (target) stimulus was presented. If the subject did not correctly indicate the target, the corresponding epoch was rejected from data analysis [25]. The electrodes located at the Pz and Oz positions in the 10–20 electrode system exhibited the most obvious P300 signals elicited by these visual stimuli.

To verify the design of our system, a commercial Neuroscan EEG acquisition system was used to collect the results for comparison [17] (Figure 9C). Neuroscan is a well-known developer of research software in the fields of neurophysiology, neuroimaging, and neurodiagnostic systems. Neuroscan products are used in approximately 40 countries in settings including universities, corporate laboratories and national research institutes. The Neuroscan SynAmps2 system, as shown in Figure 9C, synchronously records and amplifies EEG signals. The Neuroscan amplifier is a high-quality 64-channel digital EEG amplifier capable of 32-bit-precision sampling at 1000 Hz (Figure 9E). Before the EEG data were acquired, the contact impedance between each EEG electrode and the skin was calibrated to be less than 5 kΩ [17,26].

The reference electrode was mounted on the right ear lobe, and the grounding electrode was mounted on the left ear lobe. All EEG signals were preprocessed with a 200 Hz low-pass filter to remove high-frequency noise [17]. Three subjects participated in this experiment. All of the subjects were male and aged 23, 24, and 24 years old. All subjects were requested to participate in two sessions. Each session consisted of 300 trials and lasted approximately 10 min. Each subject was allowed a 5-min rest between sessions.

**Figure 10:**
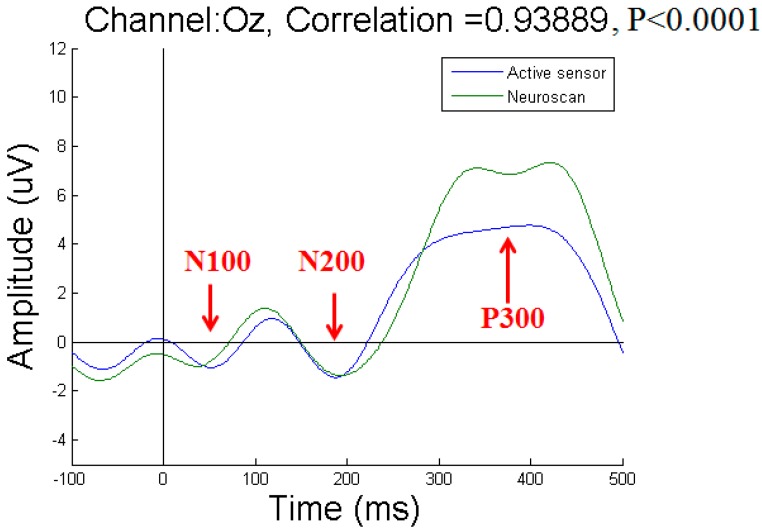
Event-related potential (ERP) results for subject 1 at Oz.

**Figure 11:**
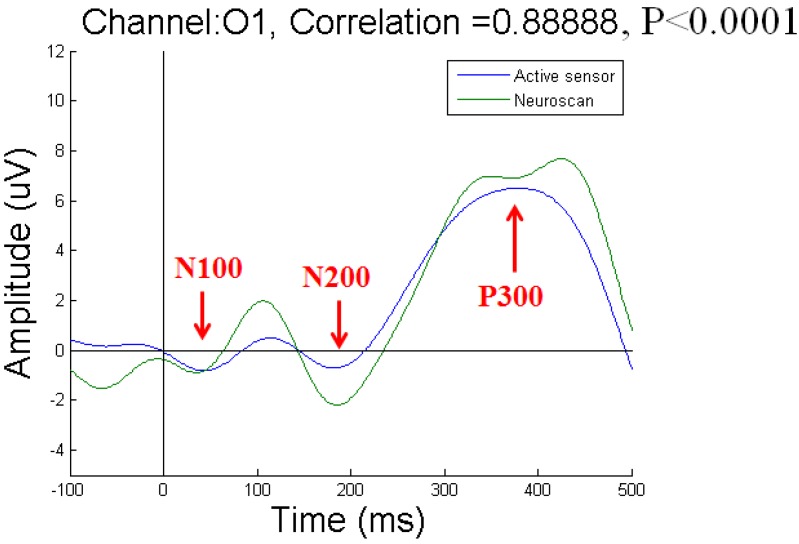
Event-related potential (ERP) results for subject 1 at O1.

**Figure 12:**
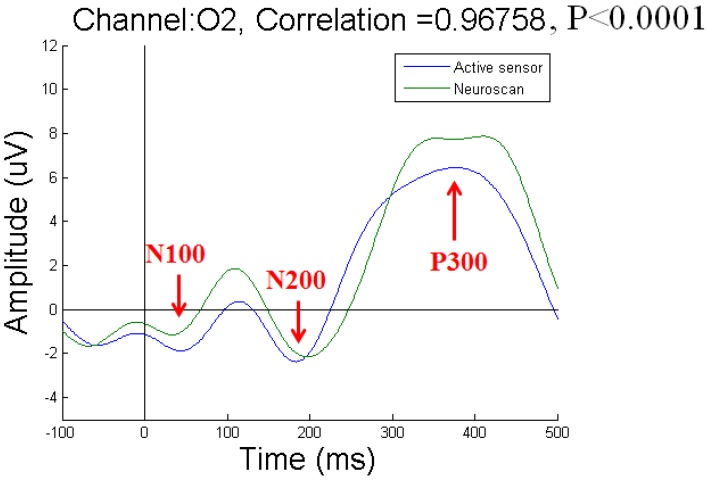
Event-related potential (ERP) results for subject 1 at O2.

To acquire P300 potentials elicited by the visual stimuli, we recorded EEG signals from three channels at the Oz, O1, and O2 positions. We compared the results obtained with our active-circuit system using dry sensors to those obtained with the Neuroscan system using wet sensors. The wet sensors were placed near the dry sensors at the Oz, O1, and O2 positions [27]. The results recorded from subject 1 at the Oz, O1, and O2 positions are shown in Figure 10, Figure 11, and Figure 12, respectively. Due to differences among individual subjects, the N100, N200, and P300 potentials were induced at different times. Overall, the N100, N200, and P300 potentials were induced at approximately 100 ms, 200 ms, and 300 ms, respectively, after the subjects were stimulated by the visual target stimulus. By comparing the results obtained with the active-circuit system to those obtained with the Neuroscan system, we could easily evaluate the performance of our developed system. The results showed good correlation between the results obtained with the two systems at the time points corresponding to the N100, N200, and P300 potentials.

As seen from the results of the ERP experiment, when compared with the commercial Neuroscan EEG acquisition system, the active-circuit system demonstrated stable reliability. We hope that this active-circuit EEG acquisition system can be beneficial to both healthy and disabled subjects in daily life in the near future [28]. Although our system shows great performance and high correlation with the Neuroscan EEG system, some aspects still warrant improvement, including the robustness and reliability of flexible electronics for long-term use.

## 4. Conclusions

With the aim of designing a light, small-sized EEG acquisition system, we fabricated a wearable, portable active-circuit system. By amplifying, filtering, and digitizing the EEG signals near the scalp, we sought to acquire clean EEG signals for transmission to the back-end system. The 8-channel active-circuit EEG acquisition system demonstrated high performance in each verification experiment. We also compared our active-circuit system with a standard EEG system, i.e., the Neuroscan EEG system. The results showed a high degree of correlation between the two systems, demonstrating that the active-circuit EEG acquisition system proposed in this study is reliable and easy to use for experiments. In addition, we markedly reduced the size of the EEG signal processing module. Because of the small size of the active-circuit system, the proposed EEG acquisition system can be used in daily life as well as in the laboratory. In summary, this verified active-circuit EEG-based BCI system is truly reliable and could provide considerable daily assistance to disabled individuals. We hope that this active-circuit EEG acquisition system will be used for various types of real-life applications and will help improve the quality of human life.

## Figures and Tables

**Table 1:** Specifications of the electroencephalography (EEG) signal acquisition system.

Specification	Active-Circuit EEG Signal Acquisition System
Channel Number	8
System Voltage Supply	3 V
Gain	9752 V/V
Bandwidth	0.103~128 Hz
ADC Resolution	24 bits
Output Current	33 mA
Battery	Lithium 3.7 V; 650 mAh (20+ h)
ADC Sampling Rate	512 Hz
Size: Active Circuit + EEG Signal Processing Module	Circular (16 mm diameter) + Rectangular (36 mm × 28 mm)
